# Protecting patients, protecting healthcare workers: a review of the role of influenza vaccination

**DOI:** 10.1111/j.1466-7657.2011.00961.x

**Published:** 2011-12-02

**Authors:** T Music

**Affiliations:** 1 Policy Analyst, Influenza Vaccines, IFPMA IVSGeneva, Switzerland

**Keywords:** Coverage, Education, Guideline, Influenza, Policy, Recommendation, Reimbursement, Seasonal, Vaccine

## Abstract

MUSIC T. (2012) A review of the role the role of influenza vaccination in protecting patients, protecting healthcare workers the role of influenza vaccination. *International Nursing Review***59**, 161–167

**Aim:**

Many health authorities recommend routine influenza vaccination for healthcare workers (HCWs), and during the 2009 A (H1N1) pandemic, the World Health Organization (WHO) recommended immunization of all HCWs worldwide. As this remains an important area of policy debate, this paper examines the case for vaccination, the role of local guidelines, barriers to immunization and initiatives to increase uptake.

**Background:**

Seasonal influenza is a major threat to public health, causing up to 1 million deaths annually. Extensive evidence supports the vaccination of priority groups, including HCWs. Immunization protects HCWs themselves, and their vulnerable patients from nosocomial influenza infections. In addition, influenza can disrupt health services and impact healthcare organizations financially. Immunization can reduce staff absences, offer cost savings and provide economic benefits.

**Methods:**

This paper reviews official immunization recommendations and HCW vaccination studies, including a recent International Federation of Pharmaceutical Manufacturers and Associations (IFPMA) survey of 26 countries from each region of the world.

**Results:**

HCW immunization is widely recommended and supported by the WHO. In the IFPMA study, 88% of countries recommended HCW vaccination, and 61% supported this financially (with no correlation to country development status). Overall, coverage can be improved, and research shows that uptake may be impacted by lack of conveniently available vaccines and misconceptions regarding vaccine safety/efficacy and influenza risk.

**Conclusions:**

Many countries recommend HCW vaccination against influenza. In recent years, there has been an increased uptake rate among HCWs in some countries, but not in others. Several initiatives can increase coverage, including education, easy access to free vaccines and the use of formal declination forms. The case for HCW vaccination is clear, and in an effort to further accelerate uptake as a patient safety measure, an increasing number of healthcare organizations, particularly in the USA, are implementing mandatory immunization policies, similar to other obligatory hygiene measures. However, it would be desirable if similar high vaccination uptake rates could be achieved through voluntary procedures.

## Aim

Following the 2009 A (H1N1) influenza pandemic, professional organizations and healthcare institutions around the world are focusing on the role healthcare worker (HCW) vaccination can play in enhancing public health. In many countries, health authorities already recommend seasonal influenza vaccination for priority groups, including healthcare workers (HCWs), and the World Health Organization (WHO) encourages annual immunization where supported by national data and capacities (WHO 2005). During the recent H1N1 influenza pandemic, the WHO went further and recommended vaccination of all HCWs worldwide to protect staff and prevent potential transmission to their patients (WHO 2009a).

These recommendations may be relatively uncontroversial, but their implementation has led to much debate. A number of authorities have developed initiatives to increase uptake among HCWs, and several professional organizations, particularly in the USA, believe progress can be accelerated by introducing mandatory vaccination policies as a patient safety measure (American Academy of Pediatrics 2010; Association for Professionals in Infection Control and Epidemiology 2011; Infectious Diseases Society of America 2010; National Patient Safety Foundation 2009; Society for Healthcare Epidemiology of America 2010). This approach was also a key topic of debate at the International Council of Nurses (ICN) 2011 conference (http://www.icn2011.ch/ICN_Conf_programme.pdf; see formal debate M7 p. 23).

This paper aims to contribute to this debate by exploring the arguments for HCW vaccination, and the potential of initiatives designed to increase uptake. The paper also adds to the current evidence base by drawing on new results from a recent IFPMA survey of vaccination recommendations in countries from each region of the world.

## Background

### Impact of influenza

Seasonal influenza is a major public health challenge. The virus causes 3–5 million cases of severe illness each year (WHO 2009b), and as stated during the World Health Assembly in 2003, is responsible for up to 1 million fatalities annually (WHA 2003). HCWs, and in particular those on the front line, may be at increased risk of contracting influenza due to exposure to infected patients as well as to viruses circulating in the community. Quantifying the risk is complex, but research suggests it may be greater for HCWs than for the general population. Annual influenza attack rates range from 5% to 10% in adults (WHO 2005); rates of 11–59% have been reported in HCWs caring for patients with influenza (Salgado et al. 2002).

Influenza also presents a threat to patients, and studies show that HCWs unknowingly risk acting as vectors for the virus. For instance, in a study of an influenza outbreak in a Canadian neonatal intensive care unit, 19 patients (35%) were infected and one died. Of the 86 staff included in the study, 85% were not vaccinated against influenza. In the 4 months preceding the study, 33% of workers had influenza-like illness, with half occurring during the outbreak. In 86% of cases, HCWs had not taken time off work as a result of the illness (Cunney et al. 2000). In a Scottish study of 518 HCWs in an acute hospital, 23% tested positive for infection following a mild epidemic season. Of these workers, 59% could not recall having had influenza and 28% did not remember having any respiratory illness (Elder et al. 1996).

### Organizational impact of influenza

Influenza outbreaks can result in staff absences, disrupt health services and increase healthcare costs. In the Scottish study referenced above (Elder et al. 1996), 23% of HCWs took sick leave due to influenza-like illness in a single season. Of these, more than one third were absent due to laboratory-confirmed disease, on average of 4 days.

A study in France quantified the impact of a single influenza outbreak in a 19-bed internal medicine unit (Sartor et al. 2002). The outbreak resulted in the suspension of all emergency admissions for 11 days, the postponement of eight scheduled admissions and an average extra cost of $3798 per infected patient.

### Influenza prevention

Of the countermeasures available to prevent influenza and its potentially severe complications, the WHO considers that immunization is the most effective (WHO 2009b). Vaccination is well established, and ‘safe and effective vaccines have been available and used for more than 60 years’ (WHO 2009b). By 2010, over 40% of WHO member states had incorporated seasonal influenza vaccine into their national immunization schedules (Miller 2010). Vaccination can prevent 70–90% of influenza-specific illness in healthy adults (WHO 2009b), and the WHO reports that trivalent inactivated vaccines have an excellent safety record (WHO 2000), with mainly local reactions and transient systemic reactions in a minority of vaccinees (WHO 2005).

### Impact of vaccination on patient care

Immunization of HCWs has been associated with improvements in patient safety and decreased mortality. In a US study, increases in the level of HCW immunization corresponded to significant reductions in the number of cases of laboratory-confirmed influenza among staff and nosocomial cases in patients (Salgado et al. 2004). In a study of 20 long-term elderly care hospitals, 50.9% of HCWs were vaccinated at institutions where immunization was offered routinely, compared with 4.9% where it was not. During the influenza season, 22.4% of patients died in the hospitals that did not offer staff vaccination (uncorrected mortality), compared with 13.6% in those that provided vaccines (Carman et al. 2000). In a study in the UK (Hayward et al. 2006), residents in care homes that provided HCW vaccination (coverage rate 48.2%) experienced significantly lower mortality, influenza-like illness and related medical consultations and hospitalizations, compared with homes that did not vaccinate workers (coverage 5.9%).

### Organizational impact of vaccination

Vaccination of HCWs can reduce workplace absences (Cella et al. 2005; Saxen & Virtanen 1999), deliver economic benefits for healthcare systems (Boersma et al. 1999; Cella et al. 2005) and provide cost savings for healthcare organizations. A study in Thailand found that investigations into a single influenza outbreak in three intensive care units incurred more than 10 times the cost of vaccinating the HCWs in those units (Apisarnthanarak et al. 2008). In a study in an Italian-teaching hospital, HCW vaccination was cost saving, with the economic benefits outweighing the costs by a factor of 4.5 (Cella et al. 2005). The researchers found that the prevalence of influenza-like illness was significantly higher in unvaccinated HCWs, resulting in the loss of 64% more working days than in the vaccinated group.

## Methods

In the context of this scientific and economic background, the authors assessed the extent to which national guidelines recommended HCW immunization, and compared this with the prioritization of other ‘traditional’ risk groups, in particular the elderly and those with chronic conditions. This analysis included previously unpublished HCW data from a 26-country survey of vaccination recommendations and reimbursement criteria, which was undertaken by the IFPMA Influenza Vaccine Supply task force (IFPMA IVS) in 2010. This survey formed part of a global study into the provision of seasonal influenza vaccines (submitted for publication). The 26 surveyed nations were selected to ensure the inclusion of at least one from each WHO region, a balance between countries of different development status [based on United Nation (UN) designations], and reliable data collection from countries where information was available.

The authors then reviewed publicly available national data on the level of vaccine coverage in HCWs, and compared this with the countries' recommendations on vaccination. Finally, the authors reviewed research into barriers to HCW vaccination and activities that may increase coverage levels.

## Results

### HCW vaccination recommendations

Many countries that recommend seasonal influenza vaccination include HCWs as a target group. The MIV study group analysed the 56 countries that accounted for the majority of the world's influenza vaccine provision (The Macroepidemiology of Influenza Vaccination (MIV) Study Group 2005). The researchers found that 43 of the 56 countries (77%) recommended HCW immunization, which was similar to but slightly lower than the proportion that targeted ‘traditional’ risk groups. Forty-nine of the 56 countries prioritized the elderly (88%), 48 (86%) those with cardiopulmonary conditions and 45 (80%) those with diabetes.

A recent study of vaccination in the Pan American Health Organization region shows that of the 35 countries that administered influenza vaccine in their public health systems, 91% targeted HCWs (Ropero-Álvarez et al. 2009). This compared with 80% that targeted the elderly (based on age alone) and 69% that targeted those with chronic conditions. In the IFPMA IVS research, 88% of countries recommended annual influenza vaccination for HCWs, 96% targeted the elderly and 92% prioritized those with chronic conditions (Table 1).

**Table 1:** Development status and vaccination recommendations and reimbursement in 26 countries

*Country*	Development status*	*Vaccination recommendation*			*Vaccination reimbursement*		
			
		*Elderly*	*Chronic illness*†	*HCWs*‡	*Elderly*	*Chronic illness*†	*HCWs*‡
Australia	More	Yes	Yes	Yes	Yes	Yes	No
Austria	More	Yes	Yes	Yes	No	No	No
Brazil	Less	Yes	Yes	Yes	Yes	Yes	Yes
Canada	More	Yes	Yes	Yes	Yes	Yes	Yes
Chile	Less	Yes	Yes	Yes	Yes	Yes	Yes
China	Less	Yes	Yes	Yes	No	No	No
Croatia	More	Yes	Yes	Yes	Yes	Yes	Yes
Egypt	Less	No	No	No	–	–	–
Germany	More	Yes	Yes	Yes	Yes	Yes	Yes
Indonesia	Less	Yes	No	No	No	No	No
Italy	More	Yes	Yes	Yes	Yes	Yes	No
Japan	More	Yes	Yes	No	Yes	Yes	No
Korea (Republic)	Less	Yes	Yes	Yes	Yes	No	No
Malta	More	Yes	Yes	Yes	–	Yes	Yes
Mexico	Less	Yes	Yes	Yes	Yes	Yes	Yes
New Zealand	More	Yes	Yes	Yes	Yes	Yes	Yes
Norway	More	Yes	Yes	Yes	–	–	–
Philippines	Less	Yes	Yes	Yes	No	No	No
Poland	More	Yes	Yes	Yes	No	No	No
Singapore	Less	Yes	Yes	Yes	No	No	Yes
South Africa	Less	Yes	Yes	Yes	No	No	Yes
Sweden	More	Yes	Yes	–	Yes	Yes	–
Thailand	Less	Yes	Yes	Yes	Yes	Yes	Yes
Turkey	Less	Yes	Yes	Yes	Yes	Yes	Yes
United Kingdom	More	Yes	Yes	Yes	Yes	Yes	Yes
USA	More	Yes	Yes	Yes	Yes	Yes	Yes
		96%	92%	88%	70%	67%	61%

* Status based on UN Department of Economic and Social Affairs Population Division classification.

†Chronic pulmonary, cardiovascular and metabolic conditions.

‡Healthcare workers.

At the supranational level, seasonal influenza vaccination for HCWs is supported by WHO (WHO 2005). This support is defined by ‘national data and capacities’, and WHO states that common use of seasonal vaccine may have been restricted previously to high-risk groups in industrialized countries due to differences in health priorities and budget limitations. In this context, the IFPMA IVS research explored whether a link existed between UN-designated development status (UN Department of Economic and Social Affairs Population Division 2009) and the inclusion of HCWs in official influenza vaccination recommendations. The results show that 12 of the 26 study countries (46%) met the UN designation of ‘less developed’, and of these, 83% recommended HCW immunization. Of the 14 countries (54%) classified as ‘more developed’, 92% recommended HCW vaccination. The authors used this data to analyse whether a correlation existed, based on the anticipated impact of local development status (i.e. ‘more-developed’ countries would be anticipated to recommend HCW vaccination and ‘less-developed’ countries would not). The results show no clear correlation between the inclusion of HCWs in vaccine recommendations and national development status (positive: negative correlation = 1.3:1).

### Vaccination coverage levels for HCWs

Few comprehensive studies have been undertaken into national influenza vaccine coverage rates for HCWs. Those that exist suggest uptake is low. A recent study conducted across 27 European countries (Mereckiene et al. 2010) found that levels were generally low (although varied widely), and had fallen in many of the countries in recent years. The researchers' initial survey found that HCW vaccination rates ranged from 14% to 48% (results available from seven countries), with an average coverage of 29.7% (non-weighted mean). A survey conducted the following year found immunization rates of 13.4% to 89.4% (results available from six countries; non-weighted average 33.9%). Of the five countries that reported results in both surveys, all had experienced a fall in uptake, with an average reduction of 5.1% (non-weighted mean; range: 0.2–14%). The surveys also examined uptake levels in other target groups, including the elderly. These were higher in this group than for HCWs in seven of the eight countries with available data, with six reporting levels of more than 50%. All of the studied countries recommended vaccination for the elderly, and most vaccine recommendations (>80%) included HCWs in hospitals, long-care facilities and outpatient clinics.

A separate study conducted in 11 European countries also reported low vaccine uptake among HCWs (Blank et al. 2009). Coverage ranged from 6.4% to 26.3% across two influenza seasons (2006/2007–2007/2008), with an average of 19.2% (non-weighted). The study found higher coverage rates in the elderly (without chronic illnesses); five of the 11 countries immunized over half of this group of elderly each year, and three had coverage levels of nearly 50%.

In the USA, the authorities recommend vaccination for HCWs, as well as other risk groups, including ‘traditional’ target groups such as the elderly. Coverage levels for HCWs appear higher than in Europe, with the US Centers for Disease Control and Prevention reporting that rates rose from 44.4% in the 2006–2007 season to 49% the following year (Centers for Disease Control and Prevention 2010). By January 2010, a survey using a somewhat different methodology reported uptake of 62%. In comparison, these rates were lower than those in the elderly aged 65 years or older, which stood at 65.6% in 2006–2007, 66.3% in 2007–2008 and 65.5% the following season.

### Factors affecting vaccine coverage

A number of studies have examined specific factors that influence influenza vaccine uptake by HCWs, in particular motivators that can drive coverage as well as barriers that may discourage vaccination (Table 2). For example, researchers in Switzerland surveyed HCWs at a university children's hospital (Tapiainen et al. 2005) and found that 87% of nurses were unvaccinated, with many citing doubts about vaccine efficacy (75%) and the need for vaccination (55%). These factors were also reported by physicians, although by a smaller proportion (41% and 23%, respectively). A substantially higher percentage of physicians cited a lack of time for vaccination compared with nurses (23% vs. 5%).

**Table 2:** Vaccination barriers and motivators (Heimberger et al. 1995;Hofmann et al. 2006;Ofstead 2009;Stephenson et al. 2002)

Barriers to HCW vaccination
Perception of vaccine inefficacy
Fear of side effects
Belief vaccine should be used for people at higher risk
Do not feel at risk
Misperception of influenza illness risks and transmission to patients
Fear of injections
Lack of time
Lack of (or perceived lack of) conveniently available vaccine
Vaccination motivators for HCWs
Vaccinated previously
Easy access to free vaccine
Aged over 45 years
Understanding of influenza as a serious illness
Understanding vaccine does not cause influenza
Personal protection

### Programmes to increase vaccine coverage

A number of healthcare organizations have developed programmes to increase levels of HCW vaccination. In the Swiss study described above (Tapiainen et al. 2005), the researchers used the results to design a series of initiatives. These included educational activities (staff letters and discussions with head nurses) to address misconceptions noted in the survey, and changes to increase vaccine accessibility (additional walk-in clinics and on-ward immunization). Within 1 year (2003/2004–2004/2005), HCW immunization rates increased from 19% to 24%. Physician rates rose from 43% to 64%, with uptake among nurses remaining static (13% vs. 14%).

A study in the USA found that the use of mandatory forms requiring HCWs to formally consent to or decline vaccination in writing (or record medical contraindications) played a role in increasing coverage (Ribner et al. 2008). Other factors included public support from management, regular feedback to supervisors on vaccine uptake and T-shirts for vaccinees. All employees were eligible for free vaccination. The study found that coverage levels increased by 55% in 1 year, rising from 43% during the 2005/2006 season to 66.5% the following year when the declination form was introduced. Nurses had the lowest rate of declining vaccination (13.2%).

To further analyse policies that may influence vaccine coverage, the IFPMA study examined whether a link existed between the inclusion of HCWs in national vaccination recommendations and the provision of reimbursement. The results (Table 1) show that of the 88% of study countries that recommended HCW vaccination, 67% supported this financially. Overall, 61% of study countries provided reimbursement for HCW immunization, compared with 70% for the elderly and 67% for those with chronic illnesses. This did not appear to be determined by national wealth; there was no clear correlation between the presence of reimbursement policies for HCW vaccination and UN-designated development status (positive: negative correlation = 0.9:1).

## Conclusions

Influenza is a highly contagious infectious disease, which can result in debilitating illness in healthy adults, and potentially fatal complications in those at risk. Vaccines remain the most effective tool to prevent influenza (WHO 2009b), and have been widely available for many years. Vaccination is widely recommended for a number of target groups, with the ICN noting that ‘many institutions recommend routine influenza vaccination for health care workers’ (International Council of Nurses 2009). While the use of influenza vaccines may have been restricted to industrialized countries historically, it appears that a number of less-developed nations now support HCW vaccination.

The scientific case for immunization of HCWs is strong, with potential benefits for staff, patients and healthcare organizations. Unvaccinated HCWs are at risk of infection through community and workplace exposure, and risk transmitting influenza to vulnerable patients. HCW vaccination can protect patients as well as those vaccinated, and immunization has been associated with reductions in nosocomial infections (Salgado et al. 2004) and mortality (Carman et al. 2000; Hayward et al. 2006). Vaccination can also reduce sick leave and provide economic benefits for healthcare institutions (Cella et al. 2005).

In light of these benefits, a number of organizations have introduced initiatives to encourage HCW vaccination. Programmes that include easy access to free vaccination, education to address misconceptions about vaccines and the need for immunization, management support and the use of declination forms have been shown to increase coverage rates (Tapiainen et al. 2005; Ribner et al. 2008). These initiatives have made progress in recent years, and now a number of professional bodies are committed to further accelerating vaccine uptake. As a result, several institutions in the USA are calling for mandatory HCW vaccination to protect both workers and patients (American Academy of Pediatrics 2010; Association for Professionals in Infection Control and Epidemiology 2011; Infectious Diseases Society of America 2010; National Patient Safety Foundation 2009; Society for Healthcare Epidemiology of America 2010). While this approach remains controversial in many modern democracies, it makes an important contribution to the policy debate by placing both the professional and ethical considerations for HCW vaccination clearly in the context of ‘quality of care’.

With health authorities around the world increasingly focusing on HCW vaccination, nurses and their representative organizations have a crucial role to play in leading policy development in this area. Given the strength of the evidence supporting HCW immunization, it is important that international, national and local nursing organizations carefully review their formal policies to ensure they offer appropriate, clear and actionable guidance. These groups may consider incorporating practical advice into their guidelines to help boost HCW vaccination levels based on proven methods and current best practice, including the use of declination forms and other policy approaches designed to change the default position of non-vaccination. Indeed, it is likely that as part of this process, organizations will consider their position on mandatory vaccination.

Recently, HCW vaccination has become a topic of high profile, sometimes highly charged, debate. As one of the most important groups of HCWs, nurses have a unique opportunity to lead this discussion within their own workplaces, based on a position of scientific understanding, professional commitment and personal action.
